# Parasitic Loads in Tissues of Mice Infected with *Trypanosoma cruzi* and Treated with AmBisome

**DOI:** 10.1371/journal.pntd.0001216

**Published:** 2011-06-28

**Authors:** Sabrina Cencig, Nicolas Coltel, Carine Truyens, Yves Carlier

**Affiliations:** Laboratoire de Parasitologie, Faculté de Médecine, Université Libre de Bruxelles, Brussels, Belgium; New York University School of Medicine, United States of America

## Abstract

**Background:**

Chagas disease is one of the most important public health problems and a leading cause of cardiac failure in Latin America. The currently available drugs to treat *T. cruzi* infection (benznidazole and nifurtimox) are effective in humans when administered during months. AmBisome (liposomal amphotericin B), already shown efficient after administration for some days in human and experimental infection with *Leishmania*, has been scarcely studied in *T. cruzi* infection.

**Aims:**

This work investigates the effect of AmBisome treatment, administered in 6 intraperitoneal injections at various times during acute and/or chronic phases of mouse *T. cruzi* infection, comparing survival rates and parasitic loads in several tissues.

**Methodology:**

Quantitative PCR was used to determine parasitic DNA amounts in tissues. Immunosuppressive treatment with cyclophosphamide was used to investigate residual infection in tissues.

**Findings:**

Administration of AmBisome during the acute phase of infection prevented mice from fatal issue. Parasitaemias (microscopic examination) were reduced in acute phase and undetectable in chronic infection. Quantitative PCR analyses showed significant parasite load reductions in heart, liver, spleen, skeletal muscle and adipose tissues in acute as well as in chronic infection. An earlier administration of AmBisome (one day after parasite inoculation) had a better effect in reducing parasite loads in spleen and liver, whereas repetition of treatment in chronic phase enhanced the parasite load reduction in heart and liver. However, whatever the treatment schedule, cyclophosphamide injections boosted infection to parasite amounts comparable to those observed in acutely infected and untreated mice.

**Conclusions:**

Though AmBisome treatment fails to completely cure mice from *T. cruzi* infection, it impedes mortality and reduces significantly the parasitic loads in most tissues. Such a beneficial effect, obtained by administrating it over a short time, should stimulate studies on using AmBisome in association with other drugs in order to shorten recovery from *T. cruzi* infection.

## Author Summary

Chagas disease is a leading cause of cardiac failure and the most important parasitic disease in terms of morbidity and mortality in Latin America. After an acute parasitaemic phase, infection naturally evolves to a long chronic phase. If the currently available trypanocidal drugs, benznidazole and nifurtimox, are effective in recent infection, they have to be administered during months and induce side effects. AmBisome, an already safe patented lipid formulation of amphotericin B, has been previously shown efficient using short time administration in treating human and experimental *Leishmania* (another *Trypanosomatidae* parasite) and fungal infections. This report evaluates the effect of AmBisome in mice infected with *T. cruzi*. Besides parasitologic evaluation, quantitative PCR was used to evaluate the parasite loads in tissues. Injections of AmBisome in acute infection allowed survival of all animals and drastically reduced the parasite loads in most tissues, whereas its administration in chronic phase strongly decreased the parasite loads in heart and liver, without completely curing the animals. Such results should encourage investigations on using AmBisome in association with standard drugs in order to improve the treatment of *T. cruzi* infection.

## Introduction

Chagas disease is a leading cause of cardiac failure and the most important parasitic disease in terms of morbidity and mortality in Latin America. Its causal agent, the protozoan parasite *Trypanosoma cruzi*, belonging to the family of *Trypanosomatidae*, currently infects 8 to 10 million persons. It is usually transmitted by the faeces of bloodsucking insect vectors (*Hemiptera Reduviidae*), human-to-human by infected blood products (and solid organ transplants), and from mother-to-child [1]–[3]. Large-scale migration of Latin Americans over the last few decades has contributed to Chagas disease becoming a global health issue [4]. There is currently an increased risk of transmission via infected blood products and/or congenital transmission in non-endemic countries, particularly in United States and Europe [5]–[8]. After an acute phase, generally asymptomatic though sometimes fatal in children, the infection evolves to an asymptomatic and silent chronic phase. Decades after primary infection, 30 to 40% of infected individuals develop a symptomatic chronic either cardiac (the most frequently encountered) and/or digestive clinical form of Chagas disease (megacolon and/or megaoesophagus), responsible for an important morbi-mortality [1], [3].

The currently used trypanocidal drugs, benznidazole and nifurtimox, were developed empirically in the 1960s and 1970s, respectively. They are more effective in early acute infection than in the late well established chronic phase. Though the lack of good markers complicates the validation of parasitic cure, these drugs appear preventing progression of cardiac chronic lesions [1], [9]–[11]. However, such drugs have to be taken for 1 to 3 months and can induce severe side effects conducing to stop the treatment [12]. Thus, the chemotherapy of Chagas disease remains an unsolved problem, and alternative or novel drugs are needed. Numbers of different compounds have been assayed in a variety of ways, even though none emerged as a new efficient treatment [13], [14].

The macrolide polyene amphotericin B is known to bind to sterols of eukaryotic cell membranes, inducing alterations of cell permeability and cell death. While amphotericin can bind to the cholesterol component of mammalian cells, inducing cytotoxic effects, it has a higher affinity for ergosterol, a component of the fungal cell membrane, leading to their specific killing. To minimize the toxic side-effects of amphotericin, a liposomal formulation of this molecule named AmBisome has been developed [15], and is a current and potent treatment of invasive fungal infections with *Candida* and *Aspergillus* [16]–[18]. As trypanosomatids also present ergosterol as component of their membranes [19], AmBisome might also be effective against infections with such parasites. Clinical trials have demonstrated the high efficacy of AmBisome treatment in human visceral leishmaniasis, leading to consider it as the first-line treatment for this disease [20]–[24]. Although ergosterol is also the predominant membrane sterol of *T. cruzi* [25], [26], few data are currently available on the effect of amphotericin on this parasite. Though several studies showed its *in vitro* trypanocidal activity [27]–[33], only one report described the *in vivo* effect of four amphotericin B formulations in mice acutely infected with *T. cruzi*. This latter study showed that a single dose of 25 mg/kg of AmBisome suppresses acute infection (on the basis of blood microscopic observations), whereas other amphotericin B lipid formulations increased the survival rate but did not eradicate infection in all animals [34].

On the basis of these results and aiming to obtain more information on the efficacy of AmBisome as a potential drug for Chagas disease, we have investigated thoroughly its effect in both acute and chronic phases of mouse *T. cruzi* infection. We have tested various schemes of treatment and studied by quantitative PCR the parasitic loads in several organs known to host parasite multiplication (heart, skeletal muscle, adipose tissue, spleen and liver).

## Methods

### Mice, infection and treatments

BALB/cJ mice were obtained from Janvier (Le Genest-St-Isle, France) and were maintained in our animal facilities in compliance with the guidelines of the ULB (Université Libre de Bruxelles) Ethic Committee for the use of laboratory animals (protocol 51 approved by CEBEA, Brussels, Belgium). Mice were infected at 6 weeks-old by intra-peritoneal (*i.p.*) injection of 1,000 blood trypomastigotes of the Tulahuen strain of *T. cruzi* (genotype TcVI; [35]). Blood parasitaemias were regularly determined by microscopic examination of tail vein blood, with a detection limit of 10,000 parasites/mL [36].

Mice were treated with 6 *i.p.* injections of AmBisome (Gilead, Paris, France; 25 mg/kg) given on alternate days starting either on the first day post-inoculation (dpi 1), during the acute parasitemic phase (dpi 10), the chronic phase (dpi 45) or both phases of infection. (dpi 10 and dpi 45). The tested dose (25 mg/kg) derived from the previous report of Yardley *et al*. [34]. Schedules and doses of AmBisome treatments, as well as mouse groups, are described in Table 1. Some chronically-infected mice were submitted to cyclophosphamide (Endoxan, Baxter, Belgium) immunosuppressive treatment (4 *i.p.* injections of 200 mg/kg on alternate days) as previously described [37].

**Table 1:** Mouse groups and AmBisome treatment schedules.

Mouse Groups^a^	n	Treatment in acute phase (dpi)	Treatment in chronic phase (dpi)	Cyclophosphamide administration (dpi)
**NT**	20	-	-	-
	6	-	-	4×200 mg/kg/dose (60–66)
**TeA**	16	6×25 mg/kg/dose (1–11)	-	-
**TA**	19	6×25 mg/kg/dose (10–20)	-	-
**TAC**	10	6×25 mg/kg/dose (10–20)	6×25 mg/kg/dose (45–55)	-
	4	6×25 mg/kg/dose (10–20)	6×25 mg/kg/dose (45–55)	4×200 mg/kg/dose (60–66)
**TC**	7	-	6×25 mg/kg/dose (45–55)	-
	6	-	6×25 mg/kg/dose (45–55)	4×200 mg/kg/dose (60–66)

a Mice were either non treated (NT) or treated (T) with AmBisome during the acute (A) and/or chronic (C) phases of infection by intraperitoneal injections (*i.p.*) on alternate days. Some mice of indicated groups received cyclophosphamide administred on alternate days from dpi 60. Data in brackets indicate the first and the last post-inoculation day (dpi) of treatment.

### Blood and organ sampling

On days 21 (acute infection) or 74 post-inoculation (chronic infection), mice were bled out under gazeous anesthesia via retro-orbital puncture and blood collected in citrated microtubes. Heart, liver, spleen, thigh muscle, and white adipose tissue (dorsal subcutaneous) were harvested after thoroughly flushing the entire mouse body with PBS [38], in order to avoid contamination of collected tissues with blood parasites. Blood and tissue samples were aliquoted and stored at −80°C until DNA extraction.

### DNA extraction

Organ pieces (50 mg) were disrupted using Magna Lyser instrument (Roche Diagnostics, Brussels, Belgium) at 6,500 rpm for 50 s in Green Beads tubes (Roche Applied Science, Brussels, Belgium) containing 400 µl of DNA Tissue buffer (Mole Genetics AS, Lysaker, Norway). Then 800 µg Proteinase K (Roche Applied Science, Brussels, Belgium) were added to the disrupted samples, and incubated for 4 h at 56°C. DNA extraction was performed on 200 µL of blood or organ digested samples using GeneMole apparatus and DNA Blood/Tissue kits (Mole Genetics AS, Lysaker, Norway), and eluted in 200 µL of GeneMole Elution buffer, according to the manufacturer recommendations.

### Generation of tissue standards for PCR

The standards for the quantitative PCR (qPCR) reactions were generated from tissue homogenates of non-infected mice (50 mg of heart, liver, spleen, skeletal muscle, adipose tissue, prepared as mentioned above), to which 10^6^ *T. cruzi* culture trypomastigotes were added, as previously described [39]. DNA (from tissues spiked with parasites), extracted as mentioned above, was serially diluted with 25 µg/mL of DNA obtained from tissues without added parasites. The 10-fold diluted prepared standards contained DNA from 10^5^ to 10^−2^ parasites equivalents per 50 ng of total DNA. A standard curve was generated from these standards to determine the DNA parasitic load in organs of infected mice.

Infected blood standards were prepared by 10-fold serial dilutions of 500 µL of fresh mouse blood artificially spiked with 10^8^ *T. cruzi* trypomastigotes, as already described [40]. DNA was extracted from each dilution as described above and a standard curve ranging from 2×10^8^ to 2×10^−1^ parasites/mL (corresponding to 2×10^5^ to 2×10^−4^ parasite equivalents per assay) was generated to determine the parasitic DNA load of infected mouse blood.

### Real-Time quantitative PCR

Real-time PCR was performed using a LightCycler® 480 system (Roche Diagnostics Brussels, Belgium) according to the manufacturer's instructions. Reactions were performed in a 25 µL final volume with either 160 nM *T. cruzi* 195-bp repeat DNA specific primers (Invitrogen, Carlsbad, California) TcZ1 5′-CGAGCTCTTGCCCACACGGGTGCT-3′ and TcZ2 5′-CCTCCAAGCAGCGGATAGTTCAGG-3′ [41] or 160 nM GAPDH Forward 5′-GACTTCAACAGCAACTCCCAC-3′ and GAPDH Reverse 5′-TCCACCACCCTGTTGCTGTA-3′ (from RTPrimer Database) and Perfecta SYBRGreen SuperMix (Quanta Biosciences, Gaithersburg USA). Each PCR reaction contained 50 ng genomic tissue DNA or 1 µL of eluted blood DNA. Amplification protocol consisted in a denaturation phase at 95°C for 5′ (RampRate 4.40°C/s), then 50 cycles of amplification (95°C 3′, (RampRate 4.40°C/s), 65°C 1′ (RampRate 2.20°C/s)). Fluorescence emission was measured at the end of the elongation step. A melting curve phase program was applied with a continuous fluorescence measurement between 50°C and 95°C (RampRate 2.20°C/s). The identity of the amplified products was checked by analysis of the melting curve carried out at the end of amplification. Each LightCycler run contained 2 negative controls (no DNA added to the reaction), and each DNA sample was quantified in duplicate. Duplicate values for each DNA sample were averaged and parasite equivalent load was calculated automatically by plotting the CP values against each standard of known concentration and calculation of the linear regression line of this curve.

### Normalization of parasite loads in tissues

To normalize the amount of tissue analyzed in each PCR reaction, we choose a housekeeping gene (GAPDH) to correct the intra-sample variations of the initial sample amount, DNA recovery and/or sample loading. Normalization with an external standard was possible because the amplification of *T. cruzi* genomic and murine GAPDH sequences occurred with the same efficiency (TcZ: 1.912; GAPDH: 1.930) [39]. For normalization, the TcZ DNA value in each tissue sample was divided by the value of the murine GAPDH DNA in the same sample.

### Statistical analysis

Results were presented as means ± SEM. Comparisons of means between groups were performed using the Mann-Whitney *U*-test. To assess differences between survival curves, a long rank test of Kaplan-Meier was performed. All tests were performed using Graph Pad software (Prism 5 version 5.02).

## Results

### Comparison of qPCR estimated- and microscopically determined-parasitaemias in untreated *T. cruzi*-infected mice

**Figure 1:**
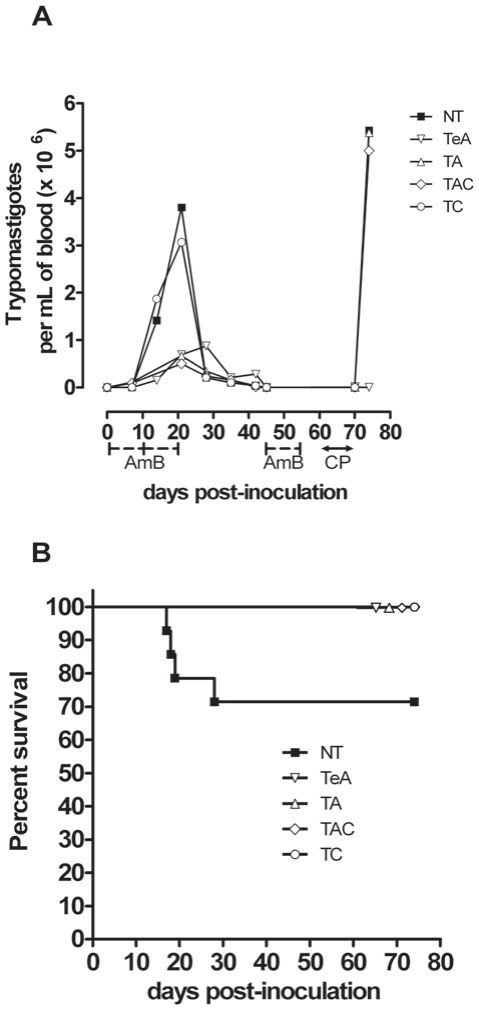
Survival and course of *T. cruzi* infection in AmBisome -treated BALB/c mice. BALB/c mice were *i.p.* inoculated with 1,000 blood trypomastigotes. NT: untreated mice; AmBisome was administred either in early acute phase (TeA), in acute phase (TA), or in chronic phase (TC). TAC mice treated by AmBisome during the acute and the chronic phases of infection. (A) Parasitaemia determined by fresh blood microscopic examination. AmB: AmBisome administration period, CP: cyclophosphamide administration period. (B) Survival curve of *T. cruzi*-infected mice.

Infection of BALB/c mice with the Tulahuen strain of *T. cruzi* resulted in an acute parasitaemic phase easily detectable by standard microscopic examination from 12 to 30 days post-inoculation (dpi), peaking at 3.8±1.5×10^6^ parasites/mL on dpi 21 (NT group, Fig. 1A). This acute phase led to the death of 30% of infected animals (Fig. 1B). Afterwards, the infection evolved to a chronic phase during which blood parasites became undetectable by standard microscopic examination. However, when such chronically infected mice received the immunosuppressive cyclophosphamide drug, they displayed a drastic increase of their blood parasite levels easily detectable by microscopic examination, reaching 5.4±3.6×10^6^ parasites/mL (on day 14 after the first cyclophosphamide injection), *i.e.* levels comparable to those previously observed in acute phase (Fig. 1A).

**Figure 2:**
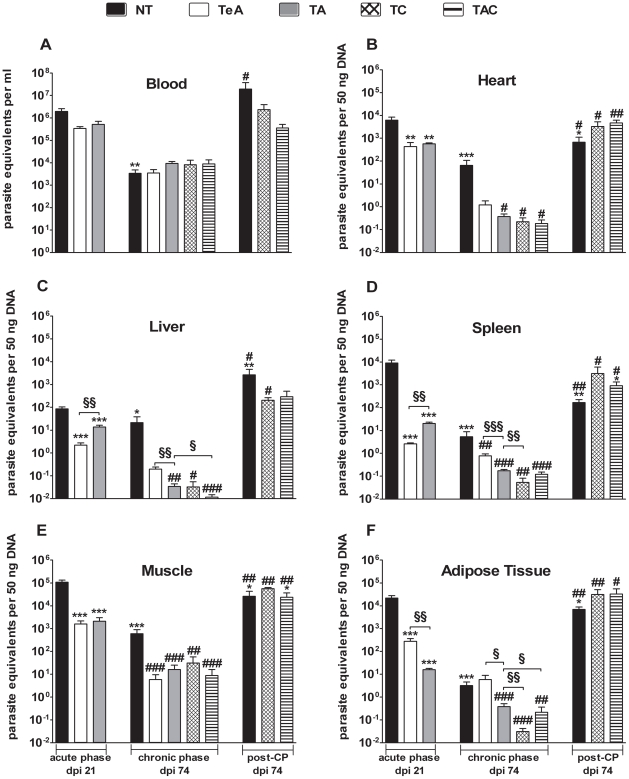
Tissue parasite amounts in AmBisome-treated mice. NT: untreated mice; AmBisome was administred either in early acute phase (TeA), in acute phase (TA), or in chronic phase (TC). TAC mice treated by AmBisome during the acute and the chronic phases of infection. Organs were collected either in acute phase (dpi 21) or in chronic phase (dpi 74) from mice having received or not cyclophosphamide (CP). Both TcZ and GAPDH sequences were quantified individually for each DNA sample. The amounts of parasite DNA in samples were expressed in parasite equivalents per mL of blood (A) or per 50 ng DNA for heart (B), liver (C), spleen (D), muscle (E) and adipose tissue (F). * denotes a significant difference with NT acute mice group, # denotes a significant difference with NT chronic mice group, § denotes a significant difference between treatments, *, #, § P<0.05; **, ##, §§ P<0.01; ***, ###, §§§ P<0.001.

Quantitative PCR determination of parasitic DNA and parasitaemia determined by microscopic observation were statistically correlated (R = 0.953, P = 0.0003) and levels estimated in the acute phase of infection (on dpi 21) were close to those determined by microscopic observation. qPCR analysis of blood samples collected on dpi 74 (chronic phase) allowed the detection of parasite DNA in all infected mice, corresponding to a mean level of 3,380±1,440 parasite equivalents/mL (NT group, Fig. 2A), *i.e.* values under the detection limit of microscopic observation. In chronically infected mice receiving cyclophosphamide, circulating parasite DNA levels drastically increased to values corresponding to 19.4±10.7×10^6^ parasite equivalents/mL (NT group, Fig. 2A).

### qPCR estimation of parasite loads in other tissues of untreated *T. cruzi*-infected mice

Levels of parasite DNA in tissues of *T. cruzi*-infected mice are shown in Fig. 2. On dpi 21 (acute phase), heart, spleen, skeletal muscle and adipose tissue displayed mean levels of parasitic DNA ranging between 6,220 and 108,000 equivalent parasites per 50 ng of total DNA, while parasite DNA amount was particularly low in liver (85.9±18.6 parasite equivalents per 50 ng of DNA). On dpi 74 (chronic phase), parasite DNA amounts were roughly reduced by 10-fold in liver, 100 fold in muscle and heart, 1,000 fold in spleen and 5,000-fold in adipose tissue as compared to acute phase. In both phases, skeletal muscle depicted the highest parasite DNA load. Again, cyclophosphamide injections induced an increase in parasite DNA levels by around 1,000 times in adipose tissue, 100-fold in hepatic tissue and only 10-fold in cardiac, spleen and skeletal muscular tissues.

### Effect of AmBisome treatment administered in acute phase on mortality and tissue parasite loads of *T. cruzi*- infected mice

As shown in Fig. 1B, administration of AmBisome to acutely infected mice (TA; dpi 10) prevented mice from fatal issue (P<0.05 as compared with untreated animals) and all treated mice survived until the end of the experiment (74 dpi). This observation, along with the fact that cell blood counts remained similar in treated and untreated animals (data not shown), suggested the absence of major toxic effects of treatment at the used dose.

As shown in Fig. 1A, microscopic observation of blood samples showed acutely infected mice treated with AmBisome significantly reducing their acute phase mean parasitaemias by 5 times, though blood parasites remained detectable at dpi 21 in all mice (TA: 6.7±0.8×10^5^ parasites/mL; NT: 3.8±1.5×10^6^ parasites/mL; P = 0.015). qPCR analyses of blood samples of treated mice showed a similar tendency to reduce by 4 times (not statistically significant) parasite DNA amounts compared to those observed in untreated mice (Fig. 2A, Table 2). By contrast, qPCR analyses of spleen and adipose tissue showed AmBisome treatment reducing their parasite loads by 449 to 1,361 as compared with NT mice, whereas such reduction was only by 6 to 51 in the other tissues (Fig. 2 B–F, Table 2).

**Table 2:** Reductions of DNA parasite loads in organs of AmBisome-treated mice.

	Acute dpi	Phase 21		Chronic dpi	Phase 74	
Tissues	TeA	TA	TeA	TA	TC	TAC
**Heart**	14	11	54	176	293	353
**Liver**	39	6	113	437	660	1835
**Spleen**	3473	449	7	31	99	45
**Muscle**	69	51	103	37	19	68
**Adipose Tissue**	77	1361	≤1	8	104	15
**Blood**	6	4	≤1	≤1	≤1	≤1

For each mouse group, reduction ratios are expressed in fold decrease compared to the mean of NT group.

We also evaluated the effect of AmBisome given during the acute phase on tissue parasite loads in chronic phase. If parasitaemia on dpi 74 remained microscopically undetectable in AmBisome-treated mice, qPCR, detected similar blood parasite DNA levels in TA mouse group and in untreated mice (NT, Fig. 2A, Table 2). By contrast, all other tissues collected on dpi 74 from TA mice exhibited a significant reduction in parasite DNA amounts compared with NT animals (by 437 in liver and 8 to 176 in the other tissues) (P<0.05, Fig. 2B–F; Table 2).

### Effect of AmBisome treatment administered in both acute and chronic phases, or in chronic phase alone, on tissue parasite loads of mice infected with *T. cruzi*

Since AmBisome treatment given during the acute phase of infection did not eliminate totally the parasites, experiments were also performed adding a second round of treatment during chronic phase to mice previously treated during the acute phase (TAC group). In parallel, other mice received injections of AmBisome only during the chronic phase of infection (TC). Treatment in chronic phase consisted on 6 *i.p.* injections of 25 mg/kg on alternate days starting on dpi 60.

TC mice showed blood parasite DNA amounts roughly similar to those of NT animals, (dpi 74, Fig. 2A), whereas they displayed a significant reduction of parasite DNA loads in all tested tissues when compared to untreated mice (dpi 74, Fig. 2B–F, P<0.05). Such reduction was stronger in liver and heart (dpi 74, Fig. 2B–C, Table 2, P<0.05). When compared to TA group, TC mice did not display major changes in tissue parasite DNA loads (dpi 74, Fig. 2B–F, Table 2).

TAC mice also presented similar blood parasite DNA amounts than NT and TA chronically infected mice (dpi 74, Fig. 2A). However, this second round of AmBisome allowed a significant reduction of parasite DNA loads in all other tested tissues when compared to NT mice (dpi 74, Fig. 2B–F, P<0.05). These parasite loads remained lower than one parasite equivalent per 50 ng tissue DNA, except in muscular tissue. This double treatment scheme significantly improved the effect previously observed in liver from the TA group (dpi 74, Fig. 2C, P<0.05), as mentioned by the calculated fold decreases (Table 2).

### Effect of AmBisome treatment administered in early acute phase on tissue parasite loads of mice infected with *T. cruzi*

We also investigated whether an earlier administration (starting on dpi 1) was able to improve the treatment efficiency of AmBisome. All treated mice (TeA) survived and displayed reduced parasitaemias (microscopic determination) compared to NT group (dpi 21, TeA: 4.88±0.82×10^5^; P<0.05). Comparison of qPCR analyses performed in TeA and NT mice (on dpi 21) showed such early treatment lowering parasite loads in all tissues (Table 2; P<0.01 except for blood), the more potent effect being observed in spleen. Interestingly, early treatment starting on dpi 1 had more pronounced effect than that starting on dpi 10 in reducing parasite loads in spleen and liver (by 6 to 8 fold; P<0.01), similar effect on muscle. However, in adipose tissue the reducing effect of the early AmBisome treatment (TeA) was less pronounced than in TA mice (P<0.01). The potential long term effect of such early treatment was also investigated by determining the tissue parasite loads on dpi 74. Excepted for adipose tissue, the latter were decreased by 7 to 113 fold as compared to NT group, as indicated by the calculated fold decreases (Table 2).

### Effect of cyclophosphamide administration in *T. cruzi-*infected mice treated with AmBisome

As reported in Fig. 1A–2A, a drastic increase of blood parasite levels was observed 14 days after the first cyclophosphamide injection (7 days after the 4^th^ injection) in all TC and TAC AmBisome-treated mice, both by microscopical and qPCR analyses, reaching parasite amounts comparable to that observed in acutely infected NT mice. We also observed that cyclophosphamide injections similarly boosted parasite DNA levels in tissues of all of these mice, as in NT mice (Fig. 2B–F). Such drastic reactivation of parasite multiplication clearly showed that animals were not completely cured from *T. cruzi* infection.

Cyclophosphamide immunosuppression test was not applied to TA and TeA mouse groups, since at the end of AmBisome treatment in acute phase, parasites were still observable in blood by standard microscopic examination, indicating they were not completely cured (see above; Fig. 1A).

## Discussion

Taken together, these results indicate that AmBisome, at the used doses (*i.p.* administration), prevents mice from fatal issue in the acute phase of infection, contributes to drastically reduce parasite loads in heart, liver, spleen, skeletal muscle and adipose tissues in acute, as well as in chronic infection, but fails to completely cure animals from *T. cruzi* infection. An earlier administration of AmBisome (on dpi 1) has a better effect in reducing parasite loads in spleen and liver in acute phase, whereas repetition of treatment in chronic phase improves the reduction of parasite loads in heart and liver.

Survival rate and parasitaemias (microscopic examination) observed in untreated mice are in agreement with our previous report using the same mouse and parasite strains [42], [43]. Our qPCR data obtained in tissues/organs from infected mice can be considered as reliable since possible contaminations by DNA from blood trypomastigotes have been drastically reduced by flushing the entire circulatory system of mice. Moreover, such data agree with those of previous works exploring *T. cruzi* infection in adipose or muscular tissues of mice by normalised qPCR [39], [44]. The high amount of parasite DNA observed in skeletal muscle and heart both in acute and chronic infection can be explained by the known muscular tropism of the used parasite strain (TcVI genotype; [45]). The lowest amount detected in liver likely relates to the involvement of this organ as a major site of immunological elimination of parasites [46]. The high amount of parasite DNA in mouse adipose tissue also confirms previous reports [44]. The more important effect of dpi 10 (TA)- *vs.* dpi 1-treatment (TeA) in reducing parasite DNA in adipose tissue might indicate a later parasite invasion of this tissue compared to others.

The comparisons of tissue parasitic loads through the course of infection indicate that the AmBisome treatment initiated in acute phase (TeA and TA) induces a global decrease of parasitic loads in all studied organs, and that this beneficial effect is long-lasting since still observed in chronic phase. The treatment given in chronic phase only (TC) has also a significant beneficial effect in reducing such organ parasitic loads. However, treatment repeated in acute and chronic phases (TAC) does not present a significant advantage over TC treatment. However, considering blood, if a significant reduction of parasitaemia (microscopic determination) can be observed after the acute phase treatments (TeA and TA), the estimations of parasite DNA in chronic phase (qPCR determination) remain similar in NT and treated mice, whatever the scheduled treatments. This latter observation might relate to a release of *T. cruzi* DNA into blood circulation, subsequent to an intra-tissue lysis of parasites by AmBisome. Such parasitic DNA release probably also occurs in acute phase. However, during this phase, high levels of inflammatory molecules, such as the serum amyloid P protein (SAP), are abundantly produced in response to *T. cruzi* infection [47]. SAP is known to capture DNA and be rapidly eliminated in liver [48], [49], which might contribute to decrease the detectable circulating DNA levels in acute, but not in chronic phase. This indicates that qPCR determination of parasitic DNA in blood does not reflect the actual parasitic load in other tissues and is not sufficient enough to appreciate the effect of a treatment.

Our results confirm that AmBisome treatment increases the survival rate of acutely infected animals, although it does not cure them, even if multiple injections of drug are used instead of only one as previously indicated [34], and if treatment is started close to inoculation date. Indeed, treated mice still displayed low levels of parasites or parasite DNA in blood and other tissues both in early (on dpi 21) as well as in late infection (on dpi 74). Our experiments with the cyclophosphamide immunosuppressive drug confirm the presence of residual tissue infection in various organs. The lack of complete curative activity of the drug might be related to a sub-estimation of its efficacy since it has been administered by *i.p.* instead of intravenous route, known to be more effective for diffusing liposome-encapsulated drugs [18], [34]. Amphotericin B is known to induce an immediate lysis of *Trypanosomatidae* parasitic protozoa whatever their strain [50], due to the interaction of its large macrolactone ring with ergosterol and other 24-alkyl sterols contained in membranes, triggering the formation of aqueous pores. Consequently, another possible explanation for the absence of complete curative effect might relate to the preferential tropism of the used parasite strain (TcVI) for muscle tissues (see above), whereas the AmBisome targeting, by its liposomal formulation, is more directed toward liver, spleen and lungs [51], [52]. So, the present results do not exclude a more pronounced curative effect using intravenous administration, or in *T. cruzi* infection with other parasite strains having different tissue distribution. Moreover, a beneficial effect of AmBisome treatment might be also expected in *T. cruzi* congenital infection, in which parasites are preferentially targeted to the liver by the fetal circulation [2], [53], since our results show the early treatment being able to reduce drastically parasite loads in liver and spleen, in addition to allowing survival of all infected animals.

Another information derived from the present study is the significant reduction of DNA parasite load observed in tissues (notably in cardiac tissue) of mice treated during the chronic phase of infection. This phase is frequently encountered in human *T. cruzi* infection and cumulative data indicate that treatment of such infected subjects with the standard benznidazole drug significantly reduce the progression of cardiac Chagas disease and increase the frequency of negative seroconversion [11]. This could stimulate studies on using association of drugs including AmBisome (requiring only some injections) in *T. cruzi-* infected patients, in order to improve and/or accelerate such beneficial evolution and definitive cure.
